# Roles of traditional medicine and traditional healers for rabies prevention and potential impacts on post-exposure prophylaxis: A literature review

**DOI:** 10.1371/journal.pntd.0010087

**Published:** 2022-01-20

**Authors:** Erin A. Beasley, Ryan M. Wallace, Andre Coetzer, Louis H. Nel, Emily G. Pieracci

**Affiliations:** 1 National Center for Emerging and Zoonotic Infectious Diseases, Centers for Disease Control and Prevention, Atlanta, Georgia, United States of America; 2 Center for Surveillance, Epidemiology and Laboratory Services, Centers for Disease Control and Prevention, Atlanta, Georgia, United States of America; 3 Global Alliance for Rabies Control, Pretoria, South Africa; 4 Department of Biochemistry, Genetics and Microbiology, Faculty of Natural and Agricultural Sciences, University of Pretoria, Pretoria, South Africa; US Department of Agriculture, UNITED STATES

## Abstract

**Introduction:**

Globally, traditional medicine is widely used to treat a variety of injuries and illnesses, including dog bites, and exposures that are risky for rabies. However, efficacy of most traditional remedies used for rabies prevention or treatment has not been demonstrated in controlled trials or proven in community-based surveys.

**Methods:**

Six databases were searched including the terms rabies, traditional treatment, traditional remedy, traditional therapy, traditional medicine, and medicinal treatment to review traditional remedies used in the prevention and treatment of rabies. In addition, published literature of rabies transmission dynamics was used to estimate statistical likelihood of dog bite victims developing rabies to provide clarity as to why traditional healers have a high apparent success rate when preventing death from rabies in victims bitten by suspected rabid dogs.

**Results:**

Literature review yielded 50 articles, including three controlled experiments, that described use of traditional remedies for rabies prevention and treatment. Traditional remedies for rabies ranged from plant- or animal-based products to spiritual rituals; however, only a few controlled mice trials were conducted, and none of these trials demonstrated efficacy in preventing or treating rabies. Risk of dying from rabies after a bite from a dog with unknown rabies status is low, 1.90% (0.05%-29.60%). Therefore, traditional healers had a 98.10% (70.40%-99.95%) apparent success rate in preventing death from suspected rabid dog bites despite inefficaciousness of herbal remedies.

**Conclusion:**

There was no universal plant species or route of administration that was consistently used for rabies prevention or treatment across countries. No traditional remedy was efficacious in the prevention or treatment of rabies in randomized controlled experiments. Understanding the cultural context under which traditional remedies are used may facilitate collaboration of traditional healers with the modern medical system to ensure timely and appropriate use of proven therapies for prevention and clinical management of rabies.

## Author summary

Traditional medicine is commonly used worldwide for a variety of ailments and diseases, including animal bite wound care and pre-clinical rabies prevention. Traditional healers often use herbal-based remedies containing local plants. Other traditional remedies include animal-based or spiritual-based methods. This literature review included plant surveys, controlled mice experiments, and community-based studies concerning rabies prevention provided by traditional healers in multiple countries. There was no consistent remedy used across the published literature, and most importantly, there were no published studies supporting effective traditional medicine methods for use in the prevention or treatment of human rabies. Our review of rabies virus transmission rates show that traditional healers have a high apparent success rate of preventing rabies deaths from dog bites, but there is no scientific or medical basis for this perception. Educating communities about proven, effective rabies prevention through post-exposure prophylaxis while understanding the cultural importance of traditional medicine is needed for promoting effective rabies prevention.

## Introduction

Dog-mediated rabies causes an estimated 59,000 human deaths worldwide annually, with the greatest burden occurring in the African and Asian continents [1]. Each human rabies death should be considered a public health failure, as rabies is nearly always a vaccine-preventable disease, and preventive health measures have been known since the time of Pasteur [2]. Rabies post-exposure prophylaxis (PEP)–as recommended in the latest World Health Organization (WHO) guidelines–consists of wound washing with soap and running water, a series of intradermal (ID) or intramuscular (IM) vaccinations, and administration of rabies immunoglobulin (RIG) when applicable [2]. Rabies PEP is effective at preventing development of disease permitting that it is administered prior to symptom onset, which is highly variable from days to multiple months [2]. When a rabies exposure is recognized, PEP should not be delayed [2].

Present-day rabies treatment can be primary or secondary. Primary healthcare interventions occur after exposure and before symptom onset. These interventions involve passive or active provision of antibody to neutralize virus in peripheral tissues or virus within the peripheral nervous system. Modern healthcare practitioners neutralize the *Rabies lyssavirus* (RABV) in persons presenting without symptoms of rabies through the application of passive immunity (via RIG) and the stimulation of the active immune response (via vaccine) [2]. When this primary intervention is properly administered prior to symptom onset, it is nearly always effective at treating a RABV exposure [2]. Secondary healthcare interventions occur after symptoms of rabies have manifested in the patient. Modern healthcare practices have rarely been associated with favorable outcomes in individuals with symptomatic rabies infection [3], and successful disease recovery has been documented in only 15 cases, although most have significant disease sequelae [4].

Resource-limited communities in rabies-endemic areas, often experience barriers to accessing rabies vaccines and/or RIG [5]. In these instances, bite victims often seek care from traditional healers for a variety of reasons: the distance to the nearest health center, high cost of the vaccine at health centers, lack of vaccine availability at health centers, lack of transportation to health centers, inability to miss work, and trust in traditional healers to cure disease [6–15]. Oftentimes, rural communities, especially those in low- and middle-income countries, seek treatment from traditional healers because they are more accessible and less expensive than modern medical practices [6,7,10–12]. According to the WHO Traditional Medicine Strategy 2014–2023, integration of traditional and complementary medicine with healthcare systems could provide increased access to healthcare services, despite the fact that traditional medicine may lack effectiveness, need more research for efficacy, and require more regulation [16].

In the modern era, traditional medicine, as defined by the WHO (2013), is “the knowledge, skill, and practices based on the theories, beliefs, and experiences indigenous to different cultures, whether explicable or not, used in the maintenance of health as well as in the prevention, diagnosis, improvement or treatment of physical and mental illness” [16 p. 15]. Traditional medicine is widely used across the globe for primary healthcare, disease prevention, and treatment of a variety of ailments and chronic diseases [16]. Traditional healers are common providers of these therapies to indigenous populations, and herbal therapy is a large component of traditional medicine [16,17]. While there is widespread use of traditional medicines, there is also limited data supporting the efficacy of these treatments for diseases such as rabies.

Depending on the community and the traditional healer’s knowledge, the remedies provided for diseases include herbal or plant-based products, animal products, or even spiritual rituals. To date, few plants have been chemically analyzed and tested in controlled trials for evidence of antiviral properties, and while some studies suggest that flavonoids, saponins, or alkaloids may have antiviral properties, none have been shown to be efficacious in either the prevention or treatment of rabies [18–22]. The belief that traditional healers or traditional medicine can prevent or cure rabies [9,10,15,23–25] is concerning given this lack of evidence-based data. However, it cannot be ignored that numerous modern medicines originated from clinical experiences in traditional medicines [26].

The primary aim of this study was to undertake an in-depth evaluation of the peer-reviewed literature assessing traditional remedies used in the prevention and treatment of rabies both to identify misconceptions in traditional medicine that could harm patients’ health but also to determine if any reported traditional medicines may hold promise for further evaluation in their rabies preventive properties. In addition, we attempted to use dog bite and rabies statistics and previously published epidemiologic data [2,27–31] to provide clarity as to why traditional healers have high apparent success rates when preventing death from rabies in victims bitten by suspected rabid dogs in countries where canine rabies is enzootic.

## Methods

### Literature review of traditional medicines pertaining to rabies treatment

An in-depth literature review was undertaken by searching the MEDLINE, Embase, Ovid Global Health, Scopus, WorldCat, and PubMed publication repositories for peer-reviewed articles not limited to human data that was published between January 1, 1950–January 1, 2021, using the terms (“rabies” OR “rabies virus”) AND (“traditional treatment,” OR “traditional remedy,” OR “traditional therapy,” OR “traditional medicine,” OR “medicinal treatment,” OR “medicinal plants,” OR “ethnobotany,” OR “ethnopharmacology”). Articles were excluded for the analysis if they were duplicates, did not mention rabies, and did not mention traditional medicine or traditional healers. Included articles mentioned both traditional medicine or traditional healers and rabies, were in English, were peer-reviewed, and had full-text available to our library sources. Two investigators performed article searches, and one of these investigators evaluated the articles by abstract and full text based on inclusion criteria. One investigator abstracted data from these articles pertaining to plant-based, animal-based, and spiritual-based traditional remedies used for rabies, or other remedies described by traditional healers for rabies. The method, frequency, duration, and rationale for remedies, if provided by the articles, were abstracted. If a remedy was specified for animal bite wound care, it was noted. Articles were evaluated by different subsections: traditional healer surveys of plant-based remedies, mice controlled experimental trials, traditional remedies for veterinary use, animal-based or spiritual-based remedies, and community knowledge, attitude, and practices (KAP) studies.

### Elucidating rabies transmission dynamics in relation to the use of traditional medicines

In an effort to demonstrate the apparent success rate of traditional healers in treating dog bite victims for rabies exposures, previously published studies were used to estimate the statistical likelihood of dog bite victims (i) being exposed to a rabid dog and (ii) subsequently developing rabies [27,29,32]. The apparent success rate of traditional or modern medicine treatment was defined as preventing death from a suspected rabid dog bite.

The following equations were used to determine the treatment’s apparent success rate under two healthcare seeking scenarios (traditional medicine only and modern medicine only):

#### Treatments for rabies exposures

Deaths_(traditional medicine)_ = N_(bites)_ * P_(rabid)_ * (1 –P_(traditional treatment success)_) * P_(death without successful treatment, ₮)_

Deaths_(modern medicine)_ = N_(bites)_ * P_(rabid)_ * (1 –P_(modern treatment success)_) * P_(death without successful treatment, ₮)_

*₮ assumes that persons who do not develop an appropriate response will have a certain probability of developing rabies progressing to death*.

#### Treatments for clinical rabies

Deaths_(traditional medicine)_ = N_(clinical rabies)_ * (1 –P_(traditional treatment success)_)

Deaths_(modern medicine)_ = N_(clinical rabies)_ * (1 –P_(modern treatment success)_)

where N_(bites)_ are the number of bite victims that seek either traditional or modern medicine, P_(rabid)_ is the probability that the biting animal was infectious for RABV, P_(traditional treatment success)_ is the probability that traditional medicines will prevent clinical rabies, P_(modern treatment success)_ is the probability that the modern medicine (PEP) will prevent rabies, P_(death without successful treatment)_ is the probability that death would occur after a bite from an infectious animal in the absence of a successful treatment. This analysis assumes that bite victims developing clinical rabies die, a baseline P_(death without successful treatment)_ is 19% as reported by Shim et. al. (2009) [26], that the rate of PEP failure is 1%, that all bite victims seeking modern medicine receive PEP and adhere to the schedule, and that bite victims would seek only one type of care. Unless otherwise stated, calculations were based on the assumptions that a theoretical population of 10,000 bite victims would seek care, with equal numbers seeking traditional medicine and modern medicine, and that 10% baseline of these bite victims had a true rabies exposure. A sensitivity analysis (ranging from 0% to 99% for each variable) was conducted in which the variables P_(rabid)_, P_(modern treatment success)_, and P_(death without successful treatment)_ were altered to reflect values likely encountered in low-endemicity and high-endemicity settings based on estimations from previous literature [2,27–31]. As P_(rabid)_ was altered for endemicity settings, this variable accounted for confirmed rabies exposures. P_(traditional treatment success)_ was assumed to be zero due to lack of published evidence of traditional remedies preventing clinical rabies. The difference between modern medicine success rates and traditional medicine apparent success rates were calculated. The apparent success rate was calculated for both traditional and modern medicine scenarios by dividing the surviving patients by the number seeking care for each respective scenario (5,000 bite victims seeking care for each scenario).

## Results

### General findings of the literature review

**Fig 1:**
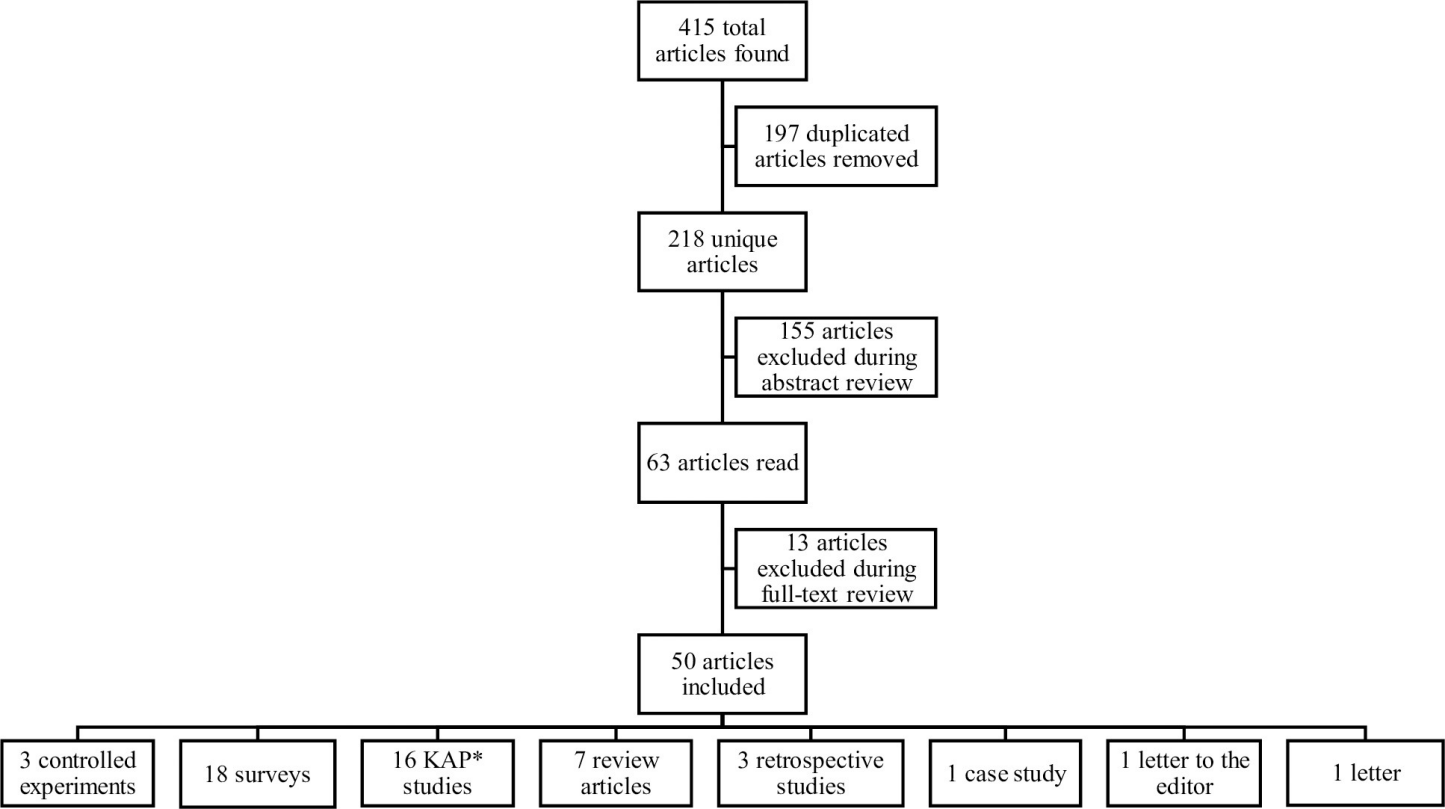
Results of literature search for articles pertaining to traditional medicines for treating rabies exposures, 1950–2021. From the 415 total articles found in the literature search, 197 articles were duplicate articles, 155 were excluded after reviewing the abstract, and 13 articles were excluded after full-text review. Fifty articles were included in this literature review. *KAP is knowledge, attitude, and practices.

In total, 415 articles were found in this literature search (Fig 1). After review, 50 articles were included in this evaluation.

### Plant remedy use described in surveys

For pre-clinical rabies prevention after dog bites, the majority of traditional remedies used were herbal. From the 50 articles included in this literature review, 15 of the 18 surveys (83.3%) listed plants as pre-clinical rabies prevention for human rabies or dog bites (S1 Appendix). Described in these 15 surveys were 41 different plants from six countries as follows: Ethiopia (20 plants, 48.8%), India (10 plants, 24.4%), Bangladesh (5 plants, 12.2%), Nigeria (4 plants, 9.8%), Bolivia (1 plant, 2.4%), China (1 plant, 2.4%). Plant use varied based on the plant part, the route of administration, and mixtures with other plants. Leaves (10 plants, 24.4%) and roots (9 plants, 22.0%) were the most common plant parts used in remedies (Table 1).

**Table 1:** Plant part used for rabies by country (N, %).

Country	Whole Plant	Leaves	Roots	Fruit	Seeds	Rhizome	Stem	Bark	Root Bark	Aerial Part	Stem Bark	Not Stated
Ethiopia	1 (16.7)	5 (50)	9 (100)	1 (33.3)	1 (25)	0 (0)	0 (0)	1 (100)	0 (0)	0 (0)	0 (0)	2 (100)
India	2 (33.3)	4 (40)	0 (0)	1 (33.3)	0 (0)	1 (50)	1 (100)	0 (0)	1 (100)	0 (0)	0 (0)	0 (0)
Bangladesh	0 (0)	0 (0)	0 (0)	1 (33.3)	3 (75)	1 (50)	0 (0)	0 (0)	0 (0)	0 (0)	0 (0)	0 (0)
Nigeria	2 (33.3)	1 (10)	0 (0)	0 (0)	0 (0)	0 (0)	0 (0)	0 (0)	0 (0)	0 (0)	1 (100)	0 (0)
Bolivia	0 (0)	0 (0)	0 (0)	0 (0)	0 (0)	0 (0)	0 (0)	0 (0)	0 (0)	1 (100)	0 (0)	0 (0)
China	1 (16.7)	0 (0)	0 (0)	0 (0)	0 (0)	0 (0)	0 (0)	0 (0)	0 (0)	0 (0)	0 (0)	0 (0)
Total	6	10	9	3	4	2	1	1	1	1	1	2

Of 6 countries included in the 15 plant surveys, leaves and roots were the most common plant parts used.

The oral route was the most common listed means of administering the remedy (21 of 41 plants, or 51.2%); however, the route of administration was not stated for 19.5% of these plants (Table 2). The plants were mostly oral remedies in the form of tea or juice, and some were mixed with milk, honey, beer, or other liquids. Most of these plants were mixed with at least one other plant species; 26 (63.4%) were used in mixtures whereas only 15 (36.6%) were the sole plant species in the remedy. Some articles described plant remedies being applied internally, externally, or topically; these terms were not further defined in these articles.

**Table 2:** Route of plants used for rabies by country (N, %).

Country	Oral	Bathing in Solution	Internal and External	Internal	Applied Externally	Topical	Not Stated
Ethiopia	14 (66.7)	1 (100)	0 (0)	0 (0)	0 (0)	0 (0)	5 (62.5)
India	4 (19)	0 (0)	0 (0)	0 (0)	4 (100)	2 (66.7)	0 (0)
Bangladesh	3 (14.3)	0 (0)	0 (0)	0 (0)	0 (0)	0 (0)	2 (25)
Nigeria	0 (0)	0 (0)	3 (100)	1 (100)	0 (0)	0 (0)	0 (0)
Bolivia	0 (0)	0 (0)	0 (0)	0 (0)	0 (0)	1 (33.3)	0 (0)
China	0 (0)	0 (0)	0 (0)	0 (0)	0 (0)	0 (0)	1 (12.5)
Total	21	1	3	1	4	3	8

Of 6 countries included in the 15 plant surveys, the oral route was the most commonly stated route of administration. The terms used in this table were described in the articles.

Duration of plant remedy use varied by plant species, and duration was often not described in the study. Additionally, there were no explained rationales for instituting a particular duration of the remedy. Likewise, dosages for the plant remedies were often not stated, but dosages may have varied according to age of the patient and other factors [6,33,34]. Overall, no plant species or route of administration was consistently used for rabies treatment across countries.

Of the 41 plants listed, there were seven families with two or more plant species. *Solanaceae* had three genera and six species represented (*Solanum indicum* L., *Solanum marginatum* L., *Solanum surattense* Burm.f., *Capsicum frutescens* L., *Datura innoxia* Mill, and *Datura metel*). *Euphorbiaceae* had three genera and four species represented (*Croton macrostachyus* Del., *Euphorbia abyssinica* J.F.Gmel., *Euphorbia neriifolia* L., and *Ricinus communis* L.). The family *Compositae* also had three genera represented (*Vernonia amygdalina* Del., *Spilanthes paniculata* Wall., and *Guizotia abyssinica* (L.F.) Cass.). Three families had two genera represented each: *Cucurbitaceae* (*Cucumis ficifolius* A. Rich. and *Zehneria scabra* [L.f,] Sond.), *Vitaceae* (*Cyphostemma cyphopetalum* (Fresen.) Desc. Ex Wild & Drummond and *Cissus cactiformis*), and *Zingiberaceae* (*Curcuma longa* L. and *Zingiber officinale*). One family, *Piperaceae*, represented one genus with two species (*Piper longum* and *Piper nigrum*).

Only four genera had more than one species included. There were three different *Solanum* species: *Solanum indicum*, *Solanum marginatum*, and *Solanum surattense* Burm.f. While these first two species were from Ethiopia and used by mixing their roots together in preparation, the third species was from India where its fruit was used as the only ingredient. Two species from the *Euphorbia* genus were included. The roots of *Euphorbia abyssinica* J.F.Gmel. were used orally in Ethiopia, and in contrast, stems of *Euphorbia neriifolia* L. were used topically in India. The *Piper* genus had two species included: *Piper nigrum* and *Piper longum*. These species were from Bangladesh and mixed together in preparation; however, *Piper nigrum*’s seeds were used whereas *Piper longum*’s fruits were used. The *Datura* genus had two species: *Datura metel* and *Datura innoxia* Mill. These species were from India and used as a leaf paste. Whereas *Datura innoxia* Mill. was used orally, *Datura metel* was applied externally and mixed with other plants.

### Controlled experiments evaluating plant remedy efficacy

Through this literature search, three *in vivo* controlled experiments were found (Table 3). Four plants were studied among these experiments: *Phytolacca dodecandra* L’Hérit., *Salix subserrata*, *Silene macroselen*, and *Datura metel* Linn. Deressa et al. (2011) selected *Salix subserrata* and *Silene macroselen* and Admasu et al. (2014) selected *Phytolacca dodecandra* L’Hérit. because of their use for traditional rabies treatment in Ethiopia [35,36]. The third study, Roy et al. (2018), selected *Datura metel* Linn. based on reported *in vitro* anti-rabies activity [37]. Three of the 41 (7.3%) unique plants listed in the general survey analysis plus *Silene macroselen* were included in one of these studies.

**Table 3:** Plants evaluated for treatment of mice inoculated with RABV in three controlled experiments.

Plant	Part of Plant Used	Extraction	Duration	Dose	Route	*Rabies lyssavirus* Strain and Inoculation	Main Findings	Country	Reference
*Phytolacca dodecandra* L’Hérit.	Leaves, Roots	Extracted in 80% ethanol	7 days	300, 600, and 1,000 mg/kg groups	Intra-gastric needle	CVS-11 diluted with PBS in masseter muscle intramuscularly	1,000 mg/kg dose of leaves plant extract significantly (p<0.05) increased survival period (mean 22.83 days) of mice versus the positive control group (mean 9.08 days)	Ethiopia	Admasu et al., 2014 [35]
*Salix subserrata*	Leaves	Extracted in chloroform, 80% methanol, or aqueous solvents by maceration	1–3 days (3 treatment groups)	80 mg/kg	Intra-gastric needle	PV diluted to 10^−3^ with sterile distilled water in gastrocnemius muscle intramuscularly	Chloroform and aqueous extracts of *S*. *subserrata* & 80% methanol extract of *S*. *macroselen* in day 1 & 3 of treatment showed significant (p<0.05) mean difference in survival time (range of 15–41 days) versus the positive control group	Ethiopia	Deressa et al., 2011 [36]
*Silene macroselen*	Roots	Extracted in chloroform, 80% methanol, or aqueous solvents by maceration	1–3 days (3 treatment groups)	80 mg/kg	Intra-gastric needle	PV diluted to 10^−3^ with sterile distilled water in gastrocnemius muscle intramuscularly	Chloroform and aqueous extracts of *S*. *subserrata* & 80% methanol extract of *S*. *macroselen* in day 1 & 3 of treatment showed significant (p<0.05) mean difference in survival time (range of 15–41 days) versus the positive control group	Ethiopia	Deressa et al., 2011 [36]
*Datura metel* Linn.	Seeds	Soxhlet, cold, and ayurvedic extracts prepared by soaking in cow urine and boiled in cow milk to make a seed powder	17 days pre-exposure group, 14 days post-exposure group	20 mg/mL for pre-exposure and post-exposure groups, 2000 mg/kg for toxicity experiment	Oral	RV CVS intracerebrally with 10 LD_50_ dose	Survival time increased by 4 days in post-exposure treatment group (14 days) compared to positive control group (10 days); 20% survival in pre-exposure treatment group at 14 days post-infection versus 0% survival in positive control group; pre- and post-exposure treatment groups had decreased log viral titer in the brain tissue titration versus the positive control group	India	Roy et al., 2018 [37]

CVS: Challenge Virus Strain. LD_50_: Median lethal dose. PBS: Phosphate-buffered saline. PV: Pasteur Virus. RV: *Rabies lyssavirus*.

Each plant was extracted in different solvents, such as ethanol, chloroform, methanol, and other aqueous solvents, and the duration of the plant remedy given to the mice varied by the study. The number of Swiss albino mice used per study was either six [35,36] or 10 mice per group [37]. While Admasu et al. (2014) and Deressa et al. (2011) used intra-gastric needle to treat mice with a plant extract, Roy et al. (2018) gave the plant extracts orally to mice [35–37]. The Admasu et al. (2014) and Roy et al. (2018) studies both used the rabies Challenge Virus Strain (CVS), while the Deressa et al. (2011) study used Pasteur Virus (PV) [35–37]. For inoculation of the RABV, Roy et al. (2018) inoculated mice intracerebrally whereas Admasu et al. (2014) and Deressa et al. (2011) inoculated mice intramuscularly (using the masseter muscle and gastrocnemius muscle, respectively) [35–37]. Only Admasu et al. (2014) indicated confirmation of rabies death by means of the direct fluorescent antibody test (FAT) of brain tissue [35].

In each study, the mice eventually died from rabies, but certain plant extractions showed increased survival time compared to control groups. Admasu et al. (2014) found that a 1,000-mg/kg dose of *Phytolacca dodecandra* L’Hérit. leaves extract increased the mean survival period of mice to 22.83 days compared to 9.08 days in the positive control group (p<0.05) [35]. Deressa et al. (2011) found a significant difference in mean survival time (p<0.05) for chloroform and aqueous extracts of *Salix subserrata* and 80% methanol of *Silene macroselen* in one day and three days of treatment; the increased mean survival time difference from the positive control group ranged from 15–41 days depending on the treatment group [36]. Roy et al. (2018) did not have statistical significance in difference of survival time between treatment groups but found increased survival time of the mice treated with *Datura metel* Linn. extract compared to the positive control group [37]. The possible acting ingredient of each extract against rabies is unknown, but Admasu et al. (2014) speculated that *Phytolacca dodecandra* L’Hérit. leaves had a ribosomal inhibiting protein (RIP) called dodecandrin that could have antiviral effects, and Roy et al. (2018) suspected an anticholinergic agent such as atropine in the *Datura metel* Linn. extract [35,37].

### Animals and other remedies used in traditional medicine for rabies

A variety of animal-based, spiritual, and other remedies have been used for pre-clinical prevention of rabies. In India, topical remedies included a charmed earthen pot or charmed metal plate with or without hymns, urine and mustard oil, cauterization with a red-hot iron or copper, copper coins, application of armlets, and cutting another body site [9,38,39]. According to Dutta (2002), the use of a charmed earthen pot or charmed metal plate failed and resulted in the death of eight people in India [39]. Other remedies in India involved insects, such as blister beetles that had been crushed and dissolved in water and drunk by the patient [40]. In Ethiopia, consuming black beetles, placing hairs from a rabid dog on a wound, and eating meat of a rabid animal were examples of animal-based remedies [23]. In a recent Ethiopian study, 48.2% of 384 respondents believed in eating a rabid animal as a remedy [41]. A documented case-patient who was exposed to rabies in Nigeria died after only receiving traditional medicine for a dog bite; the traditional medicine recommended involved eating the liver of the rabid dog and placing the dog’s hair on the bite wounds [42].

Other pre-clinical rabies prevention remedies included avoiding rivers, men avoiding contact with women, spitting on the wound with salt, isolating a patient in a dark room, and recommending to not consult a physician–with this last remedy seemingly unique to Morocco [23,43]. Spiritual rituals and sacred scriptures have been used to treat and/or diagnose rabies in Ethiopia [44]. In Ethiopia, wound care of animal bites included burning the wound with Niger oil or nug oil [35]. Traditional medicine for wound care of animal bites in the Philippines involved Tandok, or removal of rabies from a wound with an animal horn, and/or Tawak, the high-risk practice of direct suction by the mouth of a traditional healer [10]. Topical treatments included garlic or vinegar, and sometimes traditional healers sliced a wound open to increase bleeding [10].

None of the animal-based, spiritual, or other remedies have been evaluated in controlled trials, and reports of their effectiveness are purely anecdotal.

### Veterinary uses of traditional medicine for rabies

Although most of the literature described traditional medicine for human rabies patients, some mentioned traditional medicine for animals. Pre-clinical rabies prevention for dogs included oral ingestions of wajimbit beetle (head and legs removed) with milk or applying powdered seeds of wanza (*Cordia Africana*) removed of its cases and balas (*Ficus palmata*) leaf [23]. In Ethiopia, a six-plant mixture—bark of *Croton macrostachyus* Del.; roots of *Cyphostemma cyphopetalum* (Fresen.) Desc. Ex Wild & Drummond, *Solanum indicum* L., *Solanum marginatum* L.; leaves of *Juniperus procera* Endl.; seeds of *Eragrostis tef* (Zucc.) Trotter—made into water paste and given with small teff bread is a remedy for veterinary purposes [6]. Other Ethiopian plant remedies for bovine, caprine, and ovine species exposed to rabies include the leaf of *Justicia schimperiana* (Hochst. Ex Nees) T. Anderson, the root of *Phytolacca dodecandra* L’Hérit., the leaf of *Rhamnus prinoides* L’Hér., and the leaf of *Stephania abyssinica* (Quart.-Dill. & A. Rich) Walp. [45]. These four plants were individually given as a crushed mixture in water for three days or until recovery [45]. While all four plants were administered orally, the authors reported that *Phytolacca dodecandra* L’Hérit. could also be given nasally, and *Stephania abyssinica* (Quart.-Dill. & A. Rich) Walp. could also be given topically [45]. Another Ethiopian remedy for animals used holy water and sometimes herbs of a nondescript nature [44].

### Evaluation of KAP studies and retrospective case studies

Of the 50 included articles included in this literature review, 16 were KAP studies (knowledge, attitude, and practices), and three were considered retrospective studies. These studies were conducted in nine countries and evaluated use of traditional medicine for rabies and general treatment methods for dog bite victims. One KAP study from the Philippines by Amparo et al. (2018) found that 2%–8% of the 1,065 patients believed that a traditional healer is effective for medical treatment for animal-caused wounds, and approximately 10% of those patients visited a traditional healer [24]. Of those that did not seek professional medical treatment at a facility, approximately one-third visited a traditional healer [24]. The Palawan province had a low percentage of traditional healer visits due to the community PEP awareness and training traditional healers to refer patients for PEP at animal bite treatment centers [24]. A KAP study from the Philippines with 417 survey respondents reported that 50% would seek care from a traditional healer for animal bites, and 60% would prefer going to a traditional healer for treatment of an animal bite [10]. Of those who went to a traditional healer after a dog bite, 38% (63 of 165) also went to a facility for vaccines, and four respondents who saw a traditional healer for a dog bite went to a medical facility for second opinion [10]. Twenty-eight percent of community members reported they would seek help from a traditional healer if bitten [10]. Sosa (2016) proposed introducing a subsidy for indigenous people to receive rabies vaccines and having village assemblies for rabies education [10].

In Bangladesh, Ghosh et al. (2016) reported that 59% of the 3,200 respondents sought care from a traditional healer after a dog bite whereas Rumana et al. (2013) stated that 90% of dog bite victims visited a traditional healer [7,25]. From a retrospective study spanning 12 years, Ghosh et al. (2020) found 78% of 422 dog bites victims died after only receiving treatment by a traditional healer [14]. Another retrospective study in Bangladesh stated that 66.8% of 256 dog bite victims died after receiving treatment from a traditional healer [46].

In India, one KAP study reported 16.4% of 529 animal bite cases visited traditional healers or other individuals without formal medical training before visiting a healthcare facility [47]. In contrast, a study with 121 field workers in Goa, India, stated that more than 90% consulted traditional healers or faith-based healers after a dog bite [48]. Following an outbreak of rabies in cattle and a man dying after receiving traditional medicine in Punjab, India, 57.7% of study participants believed that traditional medicine could prevent rabies [15]. Examples of traditional medicine that the participants believed in the Brookes et al. (2019) study included paste (of spices, salt, mustard, oil, or ghee) on the wound, cauterization of a wound with a hot iron, powder of ground leaves and potassium permanganate on head or neck incisions, and surrounding an infected animal with fire and lime for preventing rabies exposure [15]. Among 300 households surveyed in Punjab, the application of chili (48.8%) and lime (13.1%) to wounds were described wound treatments as well as tying off a limb above a wound site (5.1%) [49]. Salve et al. (2015) described use of chili-oil paste applied directly to the bite wound in 54.6% of the 619 dog bite patients in India [8]. In addition to chili-oil paste, turmeric oil paste, herbal roots, application of armlets, and cutting at another body site were pre-clinical rabies prevention methods for 24 Indian rabies patients [9]. Of 75 households in Cambodia that experienced a relatively recent dog bite, 5.3% used traditional healers for treatment of a dog bite [50].

One KAP study from Nigeria used a retrospective clinical analysis of 84 dog bite injuries to reveal that herbal preparation of the wound was the most common treatment (60%) before arriving at the hospital [51]. In Ghana, 64.8% of 1,319 households surveyed would seek traditional treatment after a suspected rabid dog bite; some traditional methods for pre-clinical rabies prevention included use of an unspecified concoction (29.1%), use of herbs (26.5%), or consumption of the dog’s organs (9.1%) [52]. A retrospective case review in three Liberian cities reported dog bite victims self-medicating or visiting traditional healers due to remote location or having a low income [53]. In Ethiopia, 84% of survey respondents relied on traditional healers in a study by Jemberu et al. (2013) [44]. Another Ethiopian KAP study found that 81.5% of 384 respondents prefer traditional medicines for rabies treatment, but 58.7% would actively seek traditional medicine after a suspected rabid bite [41]. A survey by Bouaddi et al. (2020) found that 52.8% of 407 respondents agreed to the statement of seeking traditional medicine if bitten by a dog [43]. These KAP studies showed that across various Asian and African communities, traditional medicine and treatment by traditional healers are still widely used for dog bites or rabies.

### Rabies transmission dynamics in relation to the use of traditional medicines

**Fig 2:**
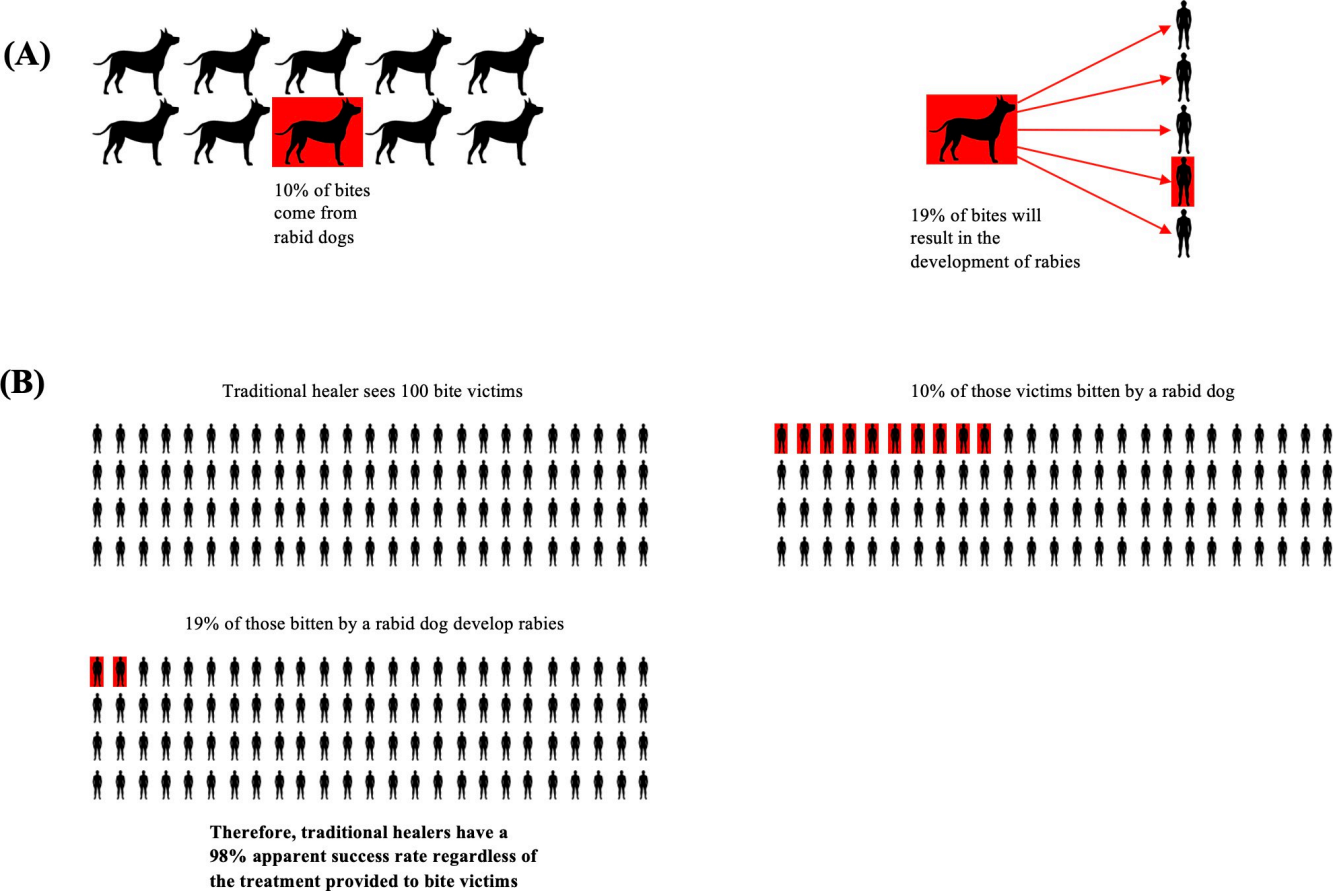
Diagram of frequencies of dog bites from rabid dogs and those bites causing clinical rabies. (A) An estimated 19% baseline value of dog bites develop into clinical rabies in humans. (B) Traditional healers treat dog bite victims, and due to the 10% estimated baseline value of bites from a rabid dog, the traditional healers have an extremely high apparent success rate of 98% of victims surviving a suspected rabid dog bite (diagram showing a population of 100).

In canine rabies enzootic countries, an estimated 10% of bites come from rabid dogs, but this risk rate can be highly variable based on the epidemiologic situation and transmission dynamics [1,28,29]. Approximately 19% of bites from rabid dogs will result in the development of rabies in the exposed individual, in the absence of PEP, and may vary by endemicity setting [27] (Table 4, Fig 2**)**. Under our assumptions previously described, a theoretical community in which 10,000 bite victims sought care equally between traditional healers and modern healthcare centers, the cohort of 5,000 people seeking traditional medicine would be expected to experience 95 (range of 2.5–1,480) rabies deaths compared to just one (range of 0–74) rabies death among the cohort seeking care at modern healthcare centers. This represents a 95-fold increase in the risk of dying from rabies among the traditional healer cohort. However, when considering the treatment’s apparent success rate of preventing death from suspected rabid dog bites, 4,905 (range of 3,520–4,977.5) people seeking care at traditional healers would survive compared to 4,999 (range of 4,926–5,000) people seeking modern medicine; this equates to an apparent success rate of 98.10% (range of 70.40%-99.95%) for traditional healers compared to 99.98% (range of 98.52%-100.00%) for modern medicine and an absolute treatment success rate difference of only 1.88% (range of 0.05%-28.12%).

**Table 4:** Probabilities of rabies deaths and treatment apparent success rates for rabies exposures by traditional medicine and modern medicine scenarios.

Variable	Baseline Value	Low-Endemicity Value	High-Endemicity Value	References
Number of Bite Victims	5,000	5,000	5,000	
P_(rabid)_	10%	1%	74%	Hampson et al., 2015. Shim et al., 2009. Wallace et al., 2015. Medley et al., 2017. Borse et al., 2018. [1,27–30]
P_(traditional treatment success)__*_	0%	0%	0%	_*_
P_(modern treatment success)_	99%	100%	95%	WHO, 2018. Shim et al., 2009. [2,27]
P_(death without successful treatment)_	19%	5%	40%	Baltazard & Ghodssi, 1954. Shim et al., 2009. [27,31]
Expected Deaths (N)–Traditional Healer	95	2.5	1,480	
Apparent Success Rate–Traditional Healer	98.10%	99.95%	70.40%	
Expected Deaths (N)–Modern Medicine	1	0	74	
Apparent Success Rate–Modern Medicine	99.98%	100.00%	98.52%	

* The treatment’s apparent success rate of traditional medicine was not changed in the sensitivity analysis since no articles provided evidence that any traditional treatment would impact the risk of dying from rabies.

The treatment’s apparent success rate for traditional healers was dependent on the probability that the biting dog had rabies and ranged from 70.40% for high-endemicity settings to 99.95% for low-endemicity settings. As no published evidence in our literature search demonstrated efficacy of traditional remedies against death from confirmed rabies, the apparent success rate of traditional healers is suspected to be similar to the rate of no treatment received by a dog bite victim. The treatment success rate for modern medicine was not dependent on the probability of rabies in the biting dog and varied only 98.52% to100.0% across the range of probabilities assessed.

## Discussion

It is impossible to confidently state that certain natural remedies applied in traditional healing practices have any beneficial effect. Foremost, there are challenges with evaluating the efficacy of traditional remedies. The plants used, preparation of the plants, duration of treatment, and route of administration vary by country and community. Many of the plants described in the surveys were wild plants, as compared to cultivated plants [33,54,55]. Dosages of plant remedies are not standardized, and plant ingredients and mixtures are usually not measured [6,33,34]. Furthermore, oftentimes the families with designated traditional healers are the only people with knowledge of herbal remedies within the community [34,55,56]. In addition, the very few published *in vivo* experimental findings suggest that the use of herbal remedies are not considered effective in the prevention or treatment of rabies.

These findings illustrate the need to comply with proven vaccine-based PEP that is both safe and effective in the prevention of rabies. However, PEP compliance will not be feasible in resource-limited countries that do not have routine access to rabies biologics and thus may rely heavily on the traditional healers within their communities. As shown here, traditional healers–and the natural medicines that they provide–could be perceived as being highly successful by community members, and it would thus most likely be impossible to negate their routine healing role. As traditional healers are often the first line of care in rural communities of developing countries, efforts should be made to incorporate them into the modern medical system in order to ensure bite victims receive timely, efficacious PEP as recommended by the WHO [16,17].

The articles in this literature review often used descriptions of a dog bite injury synonymously with rabies, even if the dog was not confirmed to be rabid. As the majority of human rabies cases result from dog bites, it is understandable how dog bite injuries would be assumed as risk for rabies. However, these terms used synonymously may misconstrue community members’ beliefs of the efficacy of traditional remedies for rabies. For example, there is likely low risk of human death after a dog bite from a non-rabid animal, even before any traditional remedy application. Most articles also did not specify if rabies treatment was for prevention of disease after a dog bite or if rabies treatment was for ameliorating clinical disease. No articles clearly described treatment for clinical rabies. Few articles specified remedies for animal-caused wound care. We assumed that articles with unspecified rabies treatment described remedies for pre-clinical prevention of rabies after an exposure to a possible rabid animal, which would correspond with modern-era primary treatment. Also, timing of traditional healer visits or use of traditional medicine was not specifically described in most articles. Additionally, confusion of other ailments with rabies may make traditional cures seem successful [23].

It should be noted that although this literature review extensively searched several databases using key terms related to rabies and traditional medicine, this review was not a systematic review, and thus some articles may have been missed. Additionally, the included articles were only in the English language, which may have excluded non-translated studies in native languages of traditional medicine communities.

The transmission of RABV from dogs to humans is an intricate process that is impacted by multiple factors, including the location and severity of the bite as well as the amount of virus deposited in the wound at the time of the injury [57]. Coupled with the knowledge that rabid animals are known to shed virus intermittently, the risk of being infected with RABV from a rabid dog bite varies from less than 10% for bites to less innervated anatomy to approximately 50% for bites to the head, face, and neck [27,31,58]. Since the incubation period is highly variable and not all dog bites result in rabies, the bite incident and potentially the type of treatment received may be long forgotten by the time that the victim eventually develops clinical disease [10]. Such factors may contribute to a falsely perceived successful intervention through traditional cure at the time of exposure [10].

Our findings of rabies transmission dynamics with traditional medicine suggest that if a traditional healer theoretically sees 5,000 bite victims over the course of a year for “rabies treatment,” fewer than 100 of those bite victims would develop rabies. This, in a sense, offers traditional healers a 98.10% (range of 70.40%-99.95%) apparent success rate of preventing death among dog bite victims, regardless of the remedy they chose to administer. We speculate that as traditional healers are respected members in the community [9,10], community members are likely to trust the efficacy and rational for treatments provided by these healers based on their apparent success rates. To combat these misconceptions, community education about rabies transmission and PEP should be provided in rural areas by local health professionals using a One Health approach.

The experiences and roles of traditional healers in the 2014–2016 West African Ebolavirus outbreak are informative. Traditional healers were sensitized to recognize signs and symptoms of Ebola disease and refer those cases to health centers for diagnosis and care [59]. Similarly, traditional healers could be taught to refer dog bite victims to health centers for PEP, and implementation of trainings between medical professionals and traditional healers may help in the timely identification of a rabies exposure and prompt administration of PEP [60]. Increased community awareness and rabies education for traditional healers can be effective in reducing rabies. In the Philippines, there was a low percentage of traditional healer visits noted in one province due to increased community awareness of the need for PEP after a dog bite [24]. Additionally, traditional healers were trained to refer patients for PEP at animal bite treatment centers [24]. Many KAP and retrospective studies included in this literature review emphasized the need for awareness of rabies prevention and PEP in the respective communities [14,41,43,46,49].

Although the three *in vivo* experiments included in this literature review showed some prolonged survival in mice receiving certain plant-based therapies while challenged with RABV, more research determining any benefit for prevention or survival from rabies with these traditional plant-based therapies is needed. The exact active chemical substance or property of the plants used in rabies traditional medicine is not known, and *in vitro* experiments of these particular plants could potentially elucidate any anti-rabies efficacy. The family *Solanaceae* had the most plant species (6 species, 14.6%) included in this literature review, and two of the four plant species used in the three experiments were from this family. It has been speculated that potential active anti-rabies ingredients in these plants may be anticholinergic alkaloids, such as atropine [18–22,37]. Others have shown *Solanaceae* plants containing active antimicrobial peptides for various pathogens, mainly bacterial or fungal [61].

While evidence for any beneficial effect of the traditional therapies identified in this review are scant, there is some biological plausibility for a theoretical beneficial result of certain therapies. The RABV can remain at the site of inoculation for days to weeks before entering the peripheral nervous system [57]. During that time, the virus is susceptible to neutralization through host antibody (vaccination), passive antibody (RIG), or phagocytosis (cellular immunity) [2]. Cellular immunity occurs prior to antibody production (humoral immunity) and can detect non-native antigens shortly after infection. Cellular immunity can be boosted through inflammation and other processes. Some of the routes of administration involve external or topical application of mixtures to the site of the bite wound. These traditional remedies may not contain compounds that directly neutralize the virus, but it is biologically plausible that they would exacerbate the host cellular immune response and theoretically improve the chances of virus recognition by the cellular immune response prior to entry into the immune-privileged nervous system. While current data-derived and evidence-based research does not support the use of any traditional remedies to treat rabies exposures, their continued use in numerous countries over centuries may be rooted in some minor health benefits, and further exploration of these biological processes should be pursued following the scientific method.

Communication between modern and traditional medicine may allow the development of a framework that could ensure more effective patient care of dog bite victims in many parts of the world. This type of campaign would need to judiciously focus on traditional healers and their communities. Therefore, it is paramount to understand and respect the cultural values of traditional remedies while advocating for timely and appropriate PEP to prevent human rabies deaths.

## Disclaimer

The findings and conclusions in this report are those of the authors and do not necessarily represent the official position of the Centers for Disease Control and Prevention.

## Supporting information

S1 Appendix Table of plants used for human rabies or dog bite treatments listed in 15 of the 18 surveys included in this literature review. (PDF)
