# Supplementary material for: Roles of traditional medicine and traditional healers for rabies prevention and potential impacts on post-exposure prophylaxis: A literature review
Source: PLoS Negl Trop Dis. 2022 Jan 20;16(1):e0010087. doi: 10.1371/journal.pntd.0010087 (PMC8775316; doi:10.1371/journal.pntd.0010087)
Supplement: S1 Appendix — (PDF) [file pntd.0010087.s001.pdf]

S1 Appendix. Table of plants used for human rabies or dog bite treatments listed in 15 of the 18 surveys included in this literature review.

Part of Plant Used	Plant	Duration/dose	Route	How Treatment is Performed	Country	Reference
**Whole Plant**	*Boswellia dalzielii* Hutch. [b]	---	Internal and external	Given as decoction and powder.	Nigeria	Ohemu et al., 2014 (p. 77) [1]
	*Calotropis gigantea** [a]	Taken at night	Oral	Pounded together with *Hybanthus enneaspermus* to make juice and mixed with coconut juice. For severe rabies, black hen blood is used instead of coconut juice.	India	Nazar et al., 2008 (p. 236) [2]
	Hibiscus rostellatus Guill. & Perr.	---	Internal	Given as decoction and powder.	Nigeria	Ohemu et al., 2014 (p. 77) [1]
	*Hybanthus enneaspermus**	Taken at night	Oral	Pounded together with *Calotropis gigantea* to make juice and mixed with coconut juice. For severe rabies, black hen blood is used instead of coconut juice.	India	Nazar et al., 2008 (p. 236) [2]
	Spilanthes paniculata Wall.	---	---	Given as decoction. Crushed.	China	Ji et al., 2004 (p. S258) [3]
	Zehneria scabra [L.f,] Sond.* [a]	One week	Bathing in solution	Fresh "leaves and a piece of root are grounded and immersed in warm water."	Ethiopia	Ragunathan & Weldegerima, 2007 (p. 202) [4]
**Leaves**	*Achyranthes aspera**	---	Applied externally	Leaf paste mixed with leaves of *Hemionitis arifolia*, leaves of *Datura metel*, and root bark of *Pongamia pinnata*.	India	Ayyanar & Ignacimuthu, 2005 (p. 251) [5]
	Afrocarpus falcatus (Thunb.) C.N. Page*	---	Oral	Fresh leaves are crushed with *Salix subserrata* Willd. and mixed with water and milk.	Ethiopia	Yinegar et al., 2008 [6]

	*Bersama abyssinica**	---	---	Mixed with roots of *Cissus cactiformis* and *Clerodendrum myricoides*.	Ethiopia	Tadesse et al., 2018 (p. 69-70) [7]
	Datura innoxia Mill.	Twice a day	Oral	"Leaf paste mixed with a pinch of sugar."	India	Das & Tag, 2006 (p. 319) [8]
	*Datura metel**	---	Applied externally	Leaf paste mixed with leaves of *Hemionitis arifolia*, leaves of *Achyranthes aspera*, and root bark of *Pongamia pinnata*.	India	Ayyanar & Ignacimuthu, 2005 (p. 251) [5]
	Juniperus procera Endl.*	---	Oral	Mixed with roots of *Cyphostemma cyphopetalum*, *Solanum indicum*, *Solanum marginatum*, seeds of *Eragrostis tef*, and bark of *Croton macrostachyus*, and the mixed powder is baked then given in water.	Ethiopia	Teklehaymanot et al., 2007 (p. 278-279) [9]
	Hemionitis arifolia (Burm.) Moore*	---	Applied externally	Leaf paste mixed with leaves of *Achyranthes aspera*, leaves of *Datura metel*, and root bark of *Pongamia pinnata*.	India	Ayyanar & Ignacimuthu, 2005 (p. 251) [5]
	Ricinus communis L.	---	Oral	Fresh leaves are pounded and squeezed then mixed with milk.	Ethiopia	Yinegar et al., 2008; Tadesse et al., 2018 (p. 72) [6,7]
	Salix subserrata Willd.*	---	Oral	Fresh leaves are crushed with *Afrocarpus falcatus* (Thunb.) C.N. Page and mixed with water and milk.	Ethiopia	Yinegar et al., 2008 [6]
	Vernonia amygdalina Del.	---	Internal and external	Macerated.	Nigeria	Ohemu et al., 2014 (p. 76) [1]
**Roots**	*Cissus cactiformis**	---	---	Mixed with leaves of *Bersama abyssinica* and roots of *Clerodendrum myricoides*.	Ethiopia	Tadesse et al., 2018 (p. 69-70) [7]

	*Clerodendrum myricoides**	---	---	Mixed with roots of *Cissus cactiformis* and leaves of *Bersama abyssinica*.	Ethiopia	Tadesse et al., 2018 (p. 69-70) [7]
	Cucumis ficifolius, A. Rich.	Twice a day for 7 days	Oral	Decoction made "by mixing 1 teaspoon root powder in 150 mL of boiling water."	Ethiopia	Ragunathan & Weldegerima, 2007 (p. 202) [4]
	Cyphostemma cyphopetalum (Fresen.) Desc. Ex Wild & Drummond*	---	Oral	Given as infusion.<br>Mixed with leaves of *Juniperus procera,* roots of *Solanum indicum*, *Solanum marginatum,* seeds of *Eragrostis teff*, and bark of *Croton macrostachyus*, and the mixed powder is baked then given in water.	Ethiopia	Teklehaymanot et al., 2007 (p.274, 278-279) [9]
	Euphorbia abyssinica J.F.Gmel.* [c]	---	Oral	Crushed root and mixed with powder of wheat or teff and dried by fire.	Ethiopia	Wubetu et al., 2017 [10]
	*Gomphocarpus stenophyllus**	---	Oral	Mixed with *Phytolacca dodecandra*, grounded and soaked in water, and filtrate "drunk on an empty stomach."	Ethiopia	Ragunathan & Weldegerima, 2007 (p. 205) [4]
	Phytolacca dodecandra L'Hérit.*	---	Oral	"Chopped roots are soaked" and "filtered suspension is drunk."<br>"Powdered with water."<br>Mixed with *Gomphocarpus stenophyllus*, grounded, soaked in water, and filtrate "drunk on an empty stomach."<br>Crushed root and drunk with honey.	Ethiopia	Esser et al., 2003 (p. 276); Teklehaymanot et al., 2007 (p. 276); Ragunathan & Weldegerima, 2007 (p. 205); Wubetu et al., 2017 [4,9-11]
	Solanum indicum L.*	---	Oral	Mixed with leaves of *Juniperus procera,* roots of *Cyphostemma cyphopetalum*, *Solanum marginatum,* seeds of *Eragrostis teff*, and bark of *Croton macrostachyus*, and the	Ethiopia	Teklehaymanot et al., 2007 (p.274, 278-279) [9]

				mixed powder is baked then given in water.		
	Solanum marginatum L.*	---	Oral	Mixed with leaves of *Juniperus procera,* roots of *Cyphostemma cyphopetalum*, *Solanum indicum,* seeds of *Eragrostis teff*, and bark of *Croton macrostachyus*, and the mixed powder is baked then given in water.	Ethiopia	Teklehaymanot et al., 2007 (p.274, 278-279) [9]
**Fruit**	*Guizotia abyssinica* (L.F.) Cass.	3 days in the mornings	Oral	Given as a cup of oil on an empty stomach.	Ethiopia	Wubetu et al., 2017 [10]
	*Piper longum**	---	Oral	Given as a powdered mixture with *Piper nigrum* seeds and *Zingiber officinale* rhizome.	Bangladesh	Sharkar et al., 2013 (p. 412) [12]
	Solanum surattense Burm.f.	Twice a day	Oral	Given peel of fruits.	India	Das & Tag, 2006 (p. 319) [8]
**Seeds**	*Capsicum frutescens* L.	---	---	---	Bangladesh	Rahmatullah et al., 2010 (p. 102) [13]
	Eragrostis tef (Zucc.) Trotter*	---	Oral	Mixed with leaves of *Juniperus procera*, roots of *Cyphostemma cyphopetalum*, *Solanum indicum*, *Solanum marginatum*, and bark of *Croton macrostachyus*, and the mixed powder is baked then given in water.	Ethiopia	Teklehaymanot et al., 2007 (p. 278-279) [9]
	Nigella sativa L.	Daily (50 g) until cure	---	Seed powder.	Bangladesh	Rahmatullah et al., 2010 (p. 102) [13]
	*Piper nigrum**	---	Oral	Given as a powdered mixture with *Piper longum* fruit and *Zingiber officinale* rhizome.	Bangladesh	Sharkar et al., 2013 (p. 412) [12]
**Rhizome**	*Curcuma longa L.** [c]	---	Topical	Applied as “turmeric with red chilli.”	India	Solanki et al., 2020 (p. 110) [14]

	*Zingiber officinale**	---	Oral	Given as a powdered mixture with *Piper longum* fruit and *Piper nigrum* seeds.	Bangladesh	Sharkar et al., 2013 (p. 412) [12]
**Stem**	*Euphorbia neriifolia L.**[c]	---	Topical	Applied "juice of Thor (cactus) with leaves of Aakado."	India	Solanki et al., 2020 (p. 110) [14]
**Bark**	*Croton macrostachyus* Del.*	---	Oral	Mixed with leaves of *Juniperus procera*, roots of *Cyphostemma cyphopetalum*, *Solanum indicum*, *Solanum marginatum*, and seeds of *Eragrostis tef*, and the mixed powder is baked then given in water.	Ethiopia	Teklehaymanot et al., 2007 (p. 278-279) [9]
**Root Bark**	*Pongamia pinnata**	---	Applied externally	Mixed with leaf paste of *Hemionitis arifolia*, *Achyranthes aspera*, and *Datura metel*.	India	Ayyanar & Ignacimuthu, 2005 (p. 251) [5]
**Aerial Part**	*Pimpinella anisum* L.	---	Topical	Given as cataplasm.	Bolivia	Fernandez et al., 2003 (p. 413) [15]
**Stem Bark**	*Khaya grandifolia*	---	Internal and external	Given as decoction.	Nigeria	Ohemu et al., 2014 (p. 77) [1]
**---**	*Brucea antidysenterica*	---	---	---	Ethiopia	Tadesse et al., 2018 (p. 72) [7]
	Scandxus multiflorus	---	---	---	Ethiopia	Tadesse et al., 2018 (p. 72) [7]

* Plant used in mixture with other plant(s).

[a] Leaves and roots from this plant are used.

[b] Leaves and stem bark from this plant are used.

[c] The other plant species used in the mixture was not specified in the preparation description.

--- No description was provided in the article.

References

1. Ohemu TL, Agunu A, Olotu PN, Ajima U, Dafam DG, Azila JJ. Ethnobotanical survey of medicinal plants used in the traditional treatment of viral infections in Jos, Plateau state, Nigeria. Int. j. med. arom. plants. 2014;4(2):74-81.
2. Nazar S, Ravikumar S, Prakash Williams G. Ethnopharmacological survey of medicinal plants along the southwest coast of India. J Herbs Spices Med Plants. 2008;14(3-4): 219–39. doi: 10.1080/10496470802598917
3. Ji H, Shengji P, Chunlin L. An ethnobotanical study of medicinal plants used by the Lisu people in Nujiang, northwest Yunnan, China. Econ Bot. 2004;58(Suppl): S253–64. doi: 10.1663/0013-0001(2004)58[S253:AESOMP]2.0.CO;2
4. Ragunathan M, Weldegerima B. Medico ethno botany; a study on the Amhara ethnic group of Gondar district of North Gondar Zone Ethiopia. Journal of Natural Remedies. 2007;7(2):200-6.
5. Ayyanar M, Ignacimuthu S. Traditional knowledge of Kani tribals in Kouthalai of Tirunelveli hills, Tamil Nadu, India. J Ethnopharmacol. 2005;102:246–55. doi: 10.1016/j.jep.2005.06.020
6. Yineger H, Yewhalaw D, Teketay D. Ethnomedicinal plant knowledge and practice of the Oromo ethnic group in southwestern Ethiopia. J Ethnobiology Ethnomedicine. 2008;4(11). doi: 10.1186/1746-4269-4-11
7. Tadesse A, Birhanu K, Firew K, Kebede M. Ethnobotanical study of medicinal plants used to treat human ailment in Guduru District of Oromia Regional State, Ethiopia. J Pharmacognosy Phytother. 2018;10(3):64–75. doi: 10.5897/JPP2018.0496
8. Das AK, Tag H. Ethnomedicinal studies of the Khamti tribe of Arunachal Pradesh. Indian Journal of Traditional Knowledge. 2006;5(3):317-22.
9. Teklehaymanot T, Giday M, Medhin G, Mekonnen Y. Knowledge and use of medicinal plants by people around Debre Libanos monastery in Ethiopia. J Ethnopharmacol. 2007;111: 271–83. doi: 10.1016/j.jep.2006.11.019
10. Wubetu M, Abula T, Dejenu G. Ethnopharmacologic survey of medicinal plants used to treat human diseases by traditional medical practitioners in Dega Damot district, Amhara, Northwestern Ethiopia. BMC Res Notes. 2017;10(157). doi: 10.1186/s13104-017-2482-3
11. Esser KB, Semagn K, Wolde-Yohannes L. Medicinal use and social status of the soap berry *endod* (*Phytolacca dodecandra*) in Ethiopia. J Ethnopharmacol. 2003;85:269–77. doi: 10.1016/S0378-8741(03)00007-2
12. Sharkar P, Rahman MM, Haque Masum GZ, Nayeem MdA, Hossen MdM, Azad AK. Ethnomedicinal importance of the plants in villages in Kushtia Sador and Mirpur Upozila, Bangladesh. J Herbs Spices Med Plants. 2013;19(4): 401–17. doi: 10.1080/10496475.2013.818606

13. Rahmatullah M, Momen A, Rahman M, Nasrin D, Hossain S, Khatun Z, et al. A randomized survey of medicinal plants used by folk medicinal practitioners in Daudkandi sub-district of Comilla district, Bangladesh. Advances in Natural and Applied Sciences. 2010;4(2):99-104.
14. Solanki D, Gadhvi K, Zala J, Vyas S. Potential of someplant speciesused as ethnomedicine growinga round Sasan Gir, Gujarat, India. Med Plnts Int Jrnl Phyt Rela Ind. 2020;12(1):105-13. doi: 10.5958/0975-6892.2020.00014.3
15. Fernandez EC, Sandi YE, Kokoska L. Ethnobotanical inventory of medicinal plants used in the Bustillo Province of the Potosi Department, Bolivia. Fitoterapia. 2003;74:407–16. doi: 10.1016/S0367-326X(03)00053-4
